# 2D and 3D Trans-vaginal Sonography to Determine Cut-offs for Ovarian Volume and Follicle Number per Ovary for Diagnosis of Polycystic Ovary Syndrome in Indian Women

**Published:** 2018

**Authors:** Kar Sujata, Samparna Swoyam

**Affiliations:** 1- Department of Obstetrics and Gynaecology, Kar Clinic and Hospital, Bhubaneswar, India; 2- Department of Radiology, Dnyandeo Yashwantrao, Patil Hospital, Mumbai, India

**Keywords:** Ovarian volume, PCOS, Polycystic ovary, Rotterdam criteria, Three dimensional ultrasound

## Abstract

**Background:**

The purpose of the study was to determine cut-off values for ovarian volume (OV) and follicle number per ovary (FNPO) in Indian women with polycystic ovary syndrome (PCOS).

**Methods:**

Eighty six PCOS women (Rotterdam criteria) and forty five matched ovulatory and normo-androgenic women were recruited. A detailed 2D and 3D trans-vaginal scan was carried out in early follicular phase (D2-D5) in all patients. Ovarian volume, follicle number per ovary, stromal volume, vascularization index (VI), vascularization flow index (VFI) and flow index (FI) were measured in PCOS and controls. Mann-Whitney test and logistic analysis using PROC LOGISTIC function of SAS^®^ (Version 9.3) were used to calculate the best cut-offs for the diagnosis of PCOS.

**Results:**

Mean ovarian volume was 13.7±5.89 and 5.06±2.44 (p<0.0001), FNPO was 19.18±6.89 and 7.13±3.51 (p<0.0001) in PCOS and controls, respectively. The cut-offs for the diagnosis of PCOS were 2D OV=6.15 *cm*^3^, 2D FNPO=12. By 3D scan, OV=7 *cm*^3^, FNPO=10, stromal volume=6 *cm*^3^, VI=4.546, VFI=2.925 and FI= 19.266. Youden’s Index (To select optimal predicted probability cut-off) was the highest for 2D FNPO (0.88786). 2D FNPO showed the highest specificity and sensitivity (AUC), 0.95238 and 0.93548, for the diagnostic accuracy of PCOS.

**Conclusion:**

2D and 3D trans-vaginal scans are equally accurate for assessment of ovarian morphology. FNPO has better diagnostic accuracy for PCOS compared to ovarian volume. Cut-off for FNPO and OV in Indian PCOS women is 12 and 6.15 *cm*^3^ by 2D, 10 and 7 *cm*^3^ by 3D trans-vaginal scan.

## Introduction

Polycystic ovary syndrome (PCOS) is the commonest endocrinopathy in reproductive age group, yet it is fraught with controversies. The diagnosis, management, pathogenesis, ethnic and racial variations, long term risks are all greatly debated. Efforts at generating consensus have been an ESHRE/ASRM initiative, leading to the 2003 Rotterdam criteria for the diagnosis of PCOS. Here, ovarian morphology was first included as the third diagnostic criteria of PCOS, along with oligo-anovulation and clinical/biochemical hyperandrogenemia. Polycystic ovarian morphology (PCOM) was defined as the presence of 12 or more follicles, measuring between 2 to 9 *mm* throughout the entire ovary (FNPO) and or an ovarian volume (OV) >10 *cm*^3^ (1, 2). PCOM as defined by Rotterdam criteria is the most accepted definition of polycystic ovary.

These thresholds were based on scant literature available. The cut-off of >10 *cm*^3^ for OV was decided on expert opinion (3), and for FNPO was based on a single study which reported 75% sensitivity and 99% specificity to distinguish between controls and PCOS (4). Since 2003, large amount of literature has been published questioning the Rotterdam criteria of polycystic ovarian morphology and its utility as a marker of PCOS.

Prevalence of PCOM in healthy, regularly menstruating women in reproductive age group has been reported as high as 25–30% (5–7). Age related variations in PCOS could influence diagnostic thresholds of PCOS (8), which is not accounted for by the Rotterdam criteria. A number of publications also report racial and ethnic differences in cut-offs to determine PCOM in women with the syndrome. Racial and ethnic differences in cut-offs of FNPO and OV have been published for Chinese and Turkish women (9, 10). There are many factors that can influence the accuracy of sonographic assessment of the ovary including observer variability, type and quality of the sonography machine, transducer frequency, route of scan (abdominal, vaginal, rectal), timing of scan with respect to menstrual cycle, and medications (Oral contraceptive pills, hormones). Most of these factors can be controlled. In our study, these confounders were overcome by having a single sonographer for all cases and controls, all scans were done in early proliferative phase and were trans-vaginal, and automated volume and follicle number calculation was performed using three dimensional software (VOCAL). Ultrasonography assessment of the ovary is dependent on the quality of the machine. Newer ultrasound imaging technologies are now available, which can make a more accurate and reproducible assessment of the follicle numbers. Based on that, AE-PCOS task force recommended that “PCOM” be defined as an FNPO 25 or more (rather than 12), an ovarian volume of 10 *ml* or more, or both. These criteria should be applied when using newer ultrasound technologies using ≥8 *MHz* transducer frequency; when image quality is not optimal, OV should be used for diagnosis (11). Counting follicles and calculating ovoid ovarian volume is also possible with a computer analysis of three dimensional (3D) imaging of the ovary. Vocal and sono AVC are brilliant computerized software tools to calculate and assess follicles and volume more accurately and with reduced inter observer variability (12, 13). However, this technique requires expensive equipment, skill to acquire and time to analyze the images. Inadequate literature exists comparing 2 dimensional and 3 dimensional imaging techniques. Current evidence lacks to suggest one over the other (12–15). Controversies of definition of PCOM led to questioning Dewailly’s idea in his 2017 review, “Diagnosis of PCOS –is it time to rethink?”; all components of PCOS, anovulation, hyperandrogenemia and PCOM need to be updated .The definition of PCOM in 2003 is now obsolete, with newer ultrasound technologies and evidence from multiple publications from different ethnic and racial groups (16).

The present study is an attempt to use 2D and3D imaging techniques in PCOS women and controls, to define polycystic ovarian morphology in women from the Indian subcontinent.

## Methods

### Subjects and study protocols:

Women presented to gynecology outpatient department with primary complain of abnormal menses and/or infertility between June 2015 to December 2016 were evaluated. Eighty-six women were diagnosed as PCOS, and forty-five normo-ovulatory, non-androgenic women were included as controls in the study. Women between the ages of 18–45 years, and all BMI groups were included in both groups. All PCOS patients were diagnosed using the following 2003 Rotterdam criteria (2 out of 3); 1. Oligo-anovulation (menstrual cycle >35 days) 2. Clinical and/or biochemical (Signs of hyperandrogenism); and 3. PCOM as identified by ultrasonography. PCOM on ultrasound was defined as follows: the presence of ≥12 follicles (FNPO) in each ovary measuring 2–9 *mm* in diameter and/or increased OV (>10 *cm*^3^). Women with endometriosis, previous ovarian or tubal surgery, any hormonal treatment over the last three months, and any abnormal ovarian cyst >10 *mm* detected during the present scan were excluded from the study. Age, obstetric history, BMI, Ferriman-Gallwey score, acne score, and anthropometric data were collected for PCOS and controls by a single technician and recorded. Controls were women who were requested to volunteer for the study. Most were hospital staff and relatives, married and fertile, with no medical or gynecological complaints, regular menstrual cycles (21–35 days) and no features of hyperandrogenemia (Acne, hirsutism based on modified FG score ≤8). All women gave written informed consent. The approval from institutional ethical committee was taken for this study (Number: KCHEC/2015/001).

### Ultrasound examination:

All participants went through a detailed transvaginal ultrasound exam by a single physician. Both controls and PCOS subjects were scanned immediately after menstruation, day 2-day 6 of menses. In case of amenorrhea, PCOS women went through progesterone withdrawal, after urine beta HCG test. An exhaustive 2D and 3D imaging of bilateral ovaries was done using a 6–12 *MHz* transvaginal volume transducer (RIC6-12-D) on a GE Voluson E8 system. Highest possible magnification was used to scan the ovaries. Real time 2D scans in long axis of the ovary from inner to outer margin were taken to determine the largest plane and its transverse section. The total number of visible follicles (FNPO) measuring 2–3 *mm* in diameter was counted manually by continuous scanning of the entire ovary. The ovarian volume (OV) was calculated using the simplified formula for prolate ellipsoid (0.5 × length × width × thickness) (4). For the 3D imaging, the 3D power Doppler image data was acquired. Vocal and sono AVC software was used to generate the data related to ovarian stromal volume, blood flow and follicle counts.

The outcome measures included FNPO, OV, ovarian stromal volume, mean grey value of the ovary, volumetric ovarian vascular indices including vascularization index (VI), flow index (FI), and vascularization flow index (VFI) and Doppler indices of the main ovarian stromal vessels.

### Hormonal assays:

Blood samples were drawn in fasting for fasting blood sugar, thyroid function tests, prolactin (both Enzyme linked fluorescence assay), total testosterone (Chemiluminescent immunoassay), lipid profiles and 75 *gm*. 2 *hr*. glucose challenge test, which is our standard protocol for diagnosis of PCOS.

### Statistical analysis:

All the statistical analysis was done using SAS^®^ Version 9.3. Mann-Whitney test and logistic regression model were used to compare the data between PCOS and control. Logistic analysis using PROC LOGISTIC function of SAS^®^ was used to calculate best cut-offs for the diagnosis of PCOS.

## Results

The anthropometric characteristics of controls and PCOS women are presented in table 1. By 2D trans-vaginal sonography, the mean OV was 13.70 ±5.89 *cm*^3^ and 5.06±2.44 *cm*^3^; FNPO was 19.18± 6.89 and 7.13±3.51, in PCOS and controls, respectively. By 3D power Doppler study, the mean OV was 11.23±4.01 and 5.72±2.83 *cm*^3^; FNPO 17.00±5.19 and 7.00±3.33, in PCOS and controls, respectively. All variables measured by 2D, 3D and 3D power Doppler study are shown in table 2. By 2D scan, OV and FNPO cut-off was 6.15 *cm*^3^ and 12. By 3D scan, OV, FNPO and stromal volume cut-off was 7 *cm*^3^, 10 and 6 *cm*^3^. A cut-off of 12 for FNPO by 2D method showed the highest specificity and sensitivity (93% and 95%). Cut-off of 7 *cm*^3^ for stromal volume by 3D scan showed 84 and 93% and cut-off of 10 *cm*^3^ for OV by 3D showed 88% and 97% (Table 3). Looking to ROC plots, AUC values for average volume, average FNPO with 2D method and average volume, average FNPO, and average stromal volume with 3D method were closer to 1. Youden’s Index for average volume, average FNPO with 2D method and average volume, average FNPO, average stromal volume with 3D method were closer to 1 (Table 3) showing the power of these tests to diagnose the disease.

**Table 1.** Baseline characteristics of both groups

	**PCOS**	**Controls**	**p-value**
**Age**	26.03±3.52	28.45±4.62	0.003
**BMI**	25.71±4.87	23.02±3.58	0.13
FG^*^ score	13.66±5.37	6.00±1.41	0.05
**WC**	87.38±8.02	89.41±9.32	0.78
**WHR**	0.91±0.06	0.85±0.06	0.01

* Ferriman Gallwey score

**Table 2.** 2D and 3D scan parameters in PCOS and controls

**2D/3D**	**Variable**	**PCOS**		**Controls**		p-value*

		**Mean**	**SD**	**Mean**	**SD**	
**2D**	Rt_VOL	14.35	6.02	5.37	2.71	<.0001
**2D**	Rt_FNPO	19.49	6.78	7.28	3.66	<.0001
**2D**	Lt_VOL	13.11	6.80	4.90	2.76	<.0001
**2D**	Lt_FNPO	18.95	8.29	6.83	3.78	<.0001
**2D**	Ave_Vol	13.70	5.89	5.06	2.44	<.0001
**2D**	Ave FNPO	19.18	6.89	7.13	3.51	<.0001
**3D**	Rt_VOL	11.48	4.67	5.88	3.04	<.0001
**3D**	Rt_FNPO	17.68	5.87	7.00	3.66	<.0001
**3D**	Lt_VOL	11.38	5.01	5.42	3.17	<.0001
**3D**	Lt_FNPO	16.58	6.50	6.52	3.25	<.0001
**3D**	Ave_Vol	11.23	4.01	5.72	2.83	<.0001
**3D**	Ave_FNPO	17.00	5.19	7.00	3.33	<.0001
**3D**	Rt_Stromal	9.90	4.05	4.72	2.07	<.0001
**3D**	Rt_VI	10.91	11.25	8.68	8.39	0.3906
**3D**	Rt_VFI	1.83	1.92	1.61	1.74	0.6391
**3D**	Rt_FI	16.84	4.71	16.56	4.79	0.8105
**3D**	Lt Stromal	9.47	3.81	4.69	2.85	<.0001
**3D**	Lt_VI	9.91	11.66	10.37	11.28	0.8729
**3D**	Lt_VFI	1.64	1.90	2.55	4.82	0.3959
**3D**	Lt_FI	16.95	5.24	16.32	5.84	0.6366
**3D**	Ave Stromal	9.47	3.39	4.75	2.07	<.0001
**3D**	Ave_VI	10.65	11.10	10.03	9.22	0.8035
**3D**	Ave_VFI	1.79	1.88	2.17	3.01	0.5681
**3D**	Ave_FI	16.84	4.70	16.35	3.17	0.5544

* p-value has been calculated using Mann-Whitney test

Note: Ave=Average of right and left

**Table 3.** Cut-offs for each scan variable for diagnosis of PCOS

	**SCAN**	**Variable**	**Cut-off**	**Probability**	**Sensitivity**	**1 - Specificity**	**Specificity**	**Youden’s Index**	**Euclidean distance**
**1**	2D	Ave_VOL	6.1515	0.69418	0.83871	0.01163	0.98837	0.82708	0.16171
**2**	2D	Ave_AFC	12	0.31696	0.93548	0.04762	0.95238	0.88786	0.08019
**3**	3D	Ave_VOL	7	0.35789	0.84000	0.06977	0.93023	0.77023	0.17455
**4**	3D	Ave_AFC	10	0.53299	0.88000	0.02326	0.97674	0.85674	0.12223
**5**	3D	Ave Stromal	6	0.33920	0.88000	0.09412	0.90588	0.78588	0.15251
**6**	3D	Ave_VI	4.546	0.23007	0.54167	0.32530	0.67470	0.21637	0.56204
**7**	3D	Ave_VFI	2.925	0.23872	0.29167	0.15854	0.84146	0.13313	0.72586
**8**	3D	Ave_FI	19.266	0.21451	0.87500	0.68293	0.31707	0.19207	0.69427

Note: Ave=Average of right and left ovary

**Table 4.** Proposed thresholds for follicle number and ovarian volume for PCOS diagnosis by different authors

	**N-PCOS**	**N-Controls**	**Proposed OV cut-off 2D**	**Proposed FNPO cut-off 2D**		**AUC SP/SE**
**Jonard et al., 2003 France**	214	112	----	≥12		0.937,99%, 75%
**Jonard et al., 2005 France**	98	57	>7 *cm*^3^	≥12		FNPO 0.956, 97%, 79% OV0.905, 68%, 91%
**Chen et al., 2008 China**	432	153	6.4 *cm*^3^	10		0V 0.898, 81%, 85.6% FNPO 0.909, 85.2%, 88.8%
**Kosus et al., 2011 Turkey**	251	65	6.43 *cm*^3^	8		OV 0.938 FNPO 0.998
**Han et al., 2017 Korea**	272	None	7.9±3.6 *cm* 36.7±3.1 *cm*^3^	14.2±4.6	13.8±4.3	----
				R&L	FNPO	
**Dewailly et al., 2011 France**	62	66	>7 *cm*^3^	≥19		FNPO 0.949, 92%, 81% OV 0.923, 89%, 87%
**Lujan et al., 2013 USA**	98	70	>10	≥26		FNPO 0.969, 94%, 85% OV 0.873, 84%, 81%
**Kar et al., 2017 India**	86	45	2D OV ≥6.15 *cms*^3^	2D FNPO ≥12		AUC0.973 Sp 95% Se93% AUC 0.961 Sp98% Se83%

## Discussion

The Rotterdam criteria of 2003 were decided based on the opinions of the majority attending the meeting rather than on any robust clinical trial evidence. The ultrasound cut-offs were based on Jonard’s study of 214 PCOS women and 112 normal controls (4), and they reported FNPO of ≥12 which offered the best compromise between specificity (99%) and sensitivity (75%). For ten years, these criteria were used for diagnosis and scientific publications. Androgen excess and polycystic ovary syndrome society (AE-PCOS) task force analyzed pertinent literature from 1985 to 2012, eliciting data from 1285 women and controls. The recommendation was to use ultrasound equipment with transducer frequency ≥ 8 *MHz*, transvaginal or transrectal route, FNPO threshold to define PCOM at ≥25 follicles. It was also noted that currently there is insufficient data to use FNPS (Follicle number in single cross-sectional plane) to define PCOM. Ovarian volume threshold of ≥10 *cm*^3^ was retained, where appropriate ultrasound equipment is not available. Dewailly (17) and Lujan’s (18) most recent studies included the comparison of detailed ovarian structure in patients with PCOS and controls and they suggested FNPO cut-offs at ≥19 and ≥26, respectively.

These conflicting results could be attributed to the fact that Dewailly et al. excluded women with PCOM from controls; however, Lujan et al. did not use this exclusion criterion. These studies mostly include white Caucasian women. Different ethnic groups from Asian populations have reported ultrasound cut-offs for diagnosis of PCOS, which are significantly different from these.

Our study comparing PCOS women to controls, had a much lower threshold for FNPO (12) and for OV (6.15C M^3^). Chen et al. (9) compared 432 PCOS (NIH criteria) women with 153 age-matched controls. High-resolution trans-vaginal/trans-rectal scans were performed in early follicular phase. They concluded that both FNPO and OV have satisfactory power for use in the diagnosis of PCOS. Cut- offs of 10 for mean FNPO and 6.4 *cm*^3^ for mean OV, obtained the best compromise between sensitivity and specificity for diagnosis of PCOS in Chinese women. Han et al. (19) studied 272 Korean women, newly diagnosed with PCOS (Rotterdam). Trans-vaginal or trans-rectal scans with 7MZs transducer were performed. They reported mean OV of 7.9 *cm*^3^ and 6.7 *cm*^3^ (Right and left ovary), mean FNPO of 14.2, and 13.8 (Right and left ovary), respectively. They concluded that in Korean nulliparous women with PCOS, OV was smaller than that in other ethnic groups.

Kosus et al. (10), studying 251 newly diagnosed PCOS women (AE-PCOS society criteria, using both chemical and biochemical criteria) and 65 controls, reported a much lower threshold for diagnosis of PCOS. Mean OV of 12.5 *cm*^3^±8.1 and 5.4 *cm*^3^±1.8 *cm*^3^ and FNPO of 9.8±2.8 and 5±1.5 in PCOS and controls were reported respectively. Cut-off for diagnosis as determined by ROC analysis was 6.43 *cm*^3^ and 8 for OV and FNPO with high sensitivity and specificity. Another Turkish study with 132 PCOS and 75 controls concluded that the optimum threshold of ovarian volume to distinguish PCOS from normal women and the mean OV in Turkish PCOS patients remains beneath the criteria by Rotterdam (20).

In 2009, Lam et al. (21) from China published 3D USG features of Chinese women with PCOS. They studied extensively 3D and 2D scan variables similar to our study. This study also compared Chinese women with previous data on Caucasian women with PCOS (22).

The strength of our study is having a very detailed 2D and 3D sonography of the ovaries using advanced software. The major limitations are small sample size of PCOS and control women. More research is needed to understand the racial differences in PCOM (If any). Since India has a highly heterogeneous population, data should be collected from all parts of the country with a much larger sample size.

## Conclusion

Sonographic assessment of the ovaries is important for the diagnosis of PCOS. Currently, controversies exist for the definition of polycystic ovarian morphology. It seems racial and ethnic differences should be investigated more in further research. Our study manifested that 2D and 3D scans are equally accurate in assessment of ovarian morphology. FNPO has better diagnostic accuracy compared to OV. There appears to be ethnic and racial differences when defining cut-offs for diagnosis of PCOS based on ovarian morphology. Asian Indian women have a much lower FNPO compared to Caucasian women.
